# Silent Invasion: Isolated Right Ventricular Metastasis from Hepatocellular Carcinoma Diagnosed by Transvenous Biopsy

**DOI:** 10.1155/cric/8832106

**Published:** 2026-10-28

**Authors:** Mounika Reddy Vadiyala, Sehajpreet Singh, Azka Naeem, Hakob Harutyunyan, Supraja Achuthanandan, David J. Epstein, Jasveen Kaur, Vijay Shetty, Simon Thein, Gerald Hollander, Jacob Shani

**Affiliations:** ^1^ Department of Internal Medicine Maimonides Medical Center Brooklyn New York USA maimonidesmed.org; ^2^ Department of Cardiology Maimonides Medical Center Brooklyn New York USA maimonidesmed.org

**Keywords:** biopsy, echocardiogram, hepatocellular carcinoma, metastases, right ventricular mass

## Abstract

Although cardiac involvement is extremely uncommon, hepatocellular carcinoma (HCC) frequently spreads to the lungs, lymph nodes, bones, and adrenal glands. The right atrium is usually affected when there is cardiac involvement because of direct invasion through the inferior vena cava. We report a 71‐year‐old man who had exertional dyspnea and a rare presentation of HCC metastasizing to the right ventricle (RV). A sizable RV mass with septal infiltration was seen on imaging. A transvenous biopsy confirmed metastatic HCC. After starting systemic therapy again, the patient reported that their clinical condition was stable. This case underscores the value of a multidisciplinary approach to diagnosis and treatment, as well as the diagnostic utility of cardiac biopsy in assessing uncommon cardiac masses.

## 1. Introduction

Hepatocellular carcinoma (HCC) is the most prevalent primary liver cancer and a leading cause of cancer‐related mortality worldwide. [1] It typically appears in the setting of cirrhosis or chronic liver disease, and its pattern of metastatic spread is well‐characterized. Although intrahepatic recurrence is most common following systemic therapy of the primary tumor or curative resection, the incidence of extrahepatic metastasis is relatively low. Common extrahepatic metastatic sites include the lungs, regional lymph nodes, bones, and adrenal glands (8%–12%) [2].

Cardiac involvement is exceedingly rare and, when present, is typically limited to the right atrium (RA) via direct extension through the inferior vena cava (IVC). Isolated right ventricular (RV) metastasis from HCC, without direct involvement of IVC or RA, is even more uncommon, with only a limited number of cases documented in the literature [3]. Additionally, such presentations pose significant diagnostic and therapeutic challenges because of their nonspecific symptoms and clinical similarities to other cardiac conditions.

Here, we report a rare case of biopsy‐confirmed isolated RV metastasis from HCC in a patient with stable disease and a remote treatment history who presented with new‐onset dyspnea. We highlight the utility of transvenous cardiac biopsy, multimodality imaging, and the necessity of a multidisciplinary approach to management.

## 2. Case Presentation

A 71‐year‐old male with a history of chronic hepatitis B‐related liver cirrhosis, primary right lobe HCC, coronary artery disease status post coronary artery bypass grafting (CABG), presented with progressively worsening chest discomfort and exertional dyspnea over several days.

His oncologic history is notable for transarterial chemoembolization (TACE) 5 years prior with subsequent remission. Two years prior, a transthoracic echocardiogram (TTE) has demonstrated normal LV function with no intracardiac masses. A surveillance abdominal MRI performed 1 year before this presentation showed no new hepatic lesions.

On the current presentation, a repeat TTE revealed a large, nonmobile, heterogeneous mass in the RV apex that measured approximately 4.4 × 2.3 cm (Figures 1 and 2). A 12‐lead electrocardiogram demonstrated sinus rhythm with nonspecific ST‐segment changes in the anterolateral leads and anterior Q waves, consistent with his prior CABG history. Cardiac magnetic resonance imaging (MRI) confirmed a 5.4 × 3.1 cm nonmobile mass occupying the RV apex and mid‐cavity with infiltration of the interventricular septum, without extension into the RA or IVC. A fluorodeoxyglucose (FDG)‐positron emission tomography (PET) scan was then performed because of the unusual location and appearance of the mass, which revealed increased FDG uptake in the RV mass as well as associated mediastinal and hilar lymph nodes, indicating a metabolically active lesion and raising strong suspicion for metastatic malignancy.

**Figure 1:**
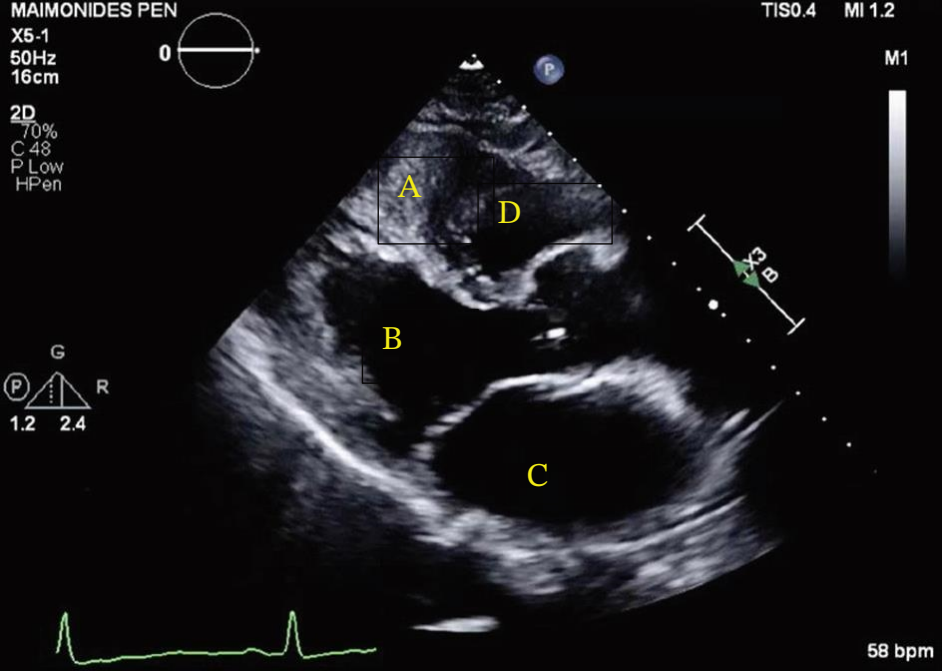
Parasternal long‐axis view showing mass encroaching the right ventricular outflow tract (RVOT). (A) Right ventricular mass. (B) Left ventricle. (C) Left atrium. (D) Right ventricular outflow tract.

**Figure 2:**
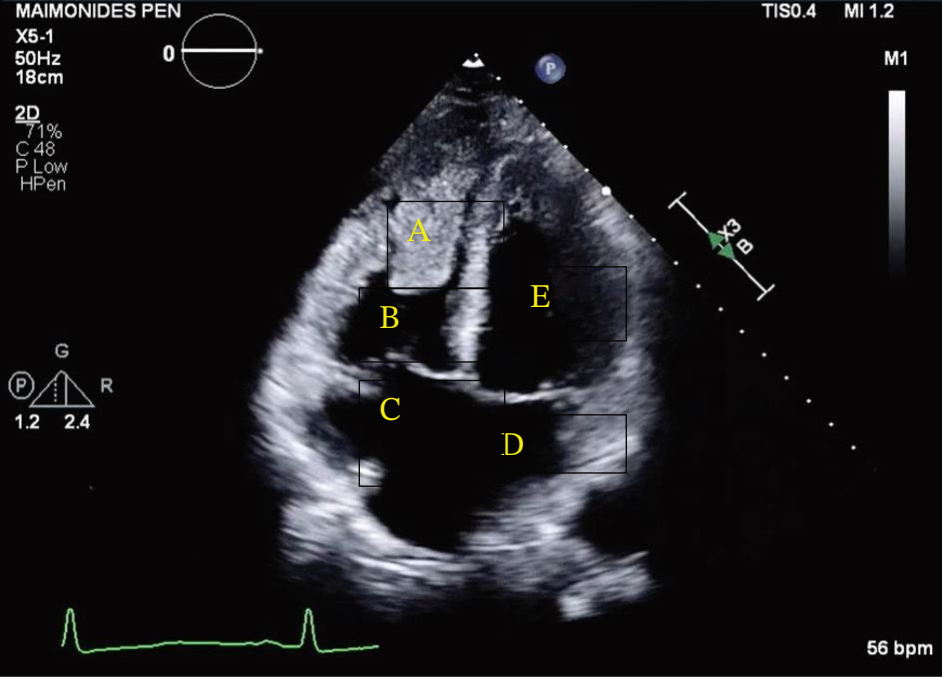
Apical 4‐chamber view showing RV mass. (A) Right ventricular mass. (B) Right ventricle. (C) Right atrium. (D) Left atrium. (E) Left ventricle.

Given the diagnostic uncertainty, especially whether the mass represented a primary cardiac tumor, thrombus or metastatic disease, and the rarity of cardiac metastasis from HCC, a multidisciplinary team comprising cardiology, oncology, and cardiothoracic surgery was involved. After careful consideration, the decision was made to proceed with an image‐guided transjugular biopsy for definitive diagnosis and management recommendations.

The RV mass was accessed for biopsy via a transvenous route through the right internal jugular (IJ) vein, under the combined guidance of TTE and fluoroscopic imaging using a snare device (Figure 3). Two tissue samples were obtained without complication and sent for analysis. Postprocedure TTE showed no pericardial effusion, and the patient was discharged in stable condition.

**Figure 3:**
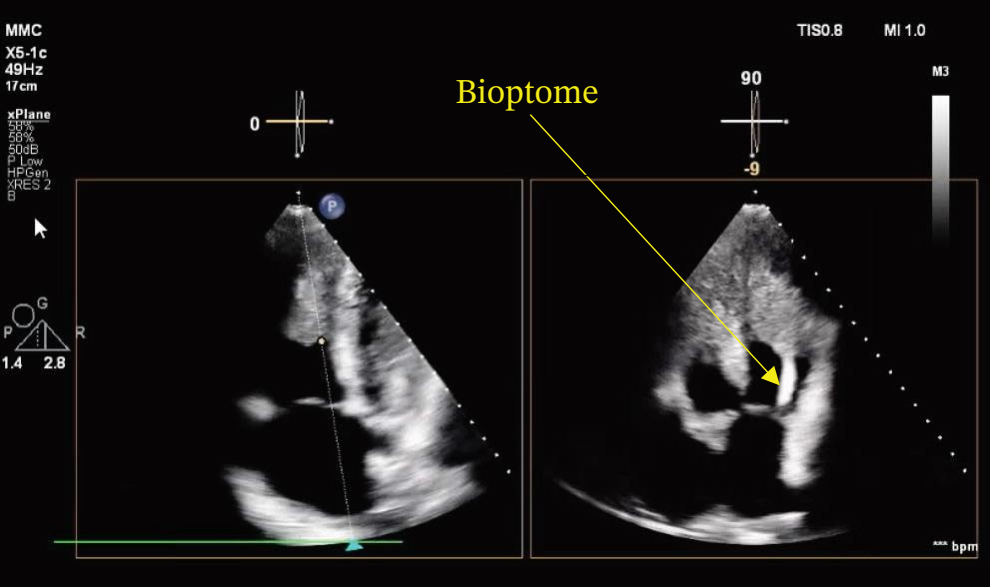
Modified apical orthogonal views showing bioptome during RV mass biopsy.

Histopathology confirmed metastatic HCC, with H&E‐stained slides revealing malignant cells with eosinophilic cytoplasm and prominent nucleoli (Figure 4). Concurrent abdominal MRI demonstrated an interval increase in the size of the hepatic lesion, consistent with recurrence.

**Figure 4:**
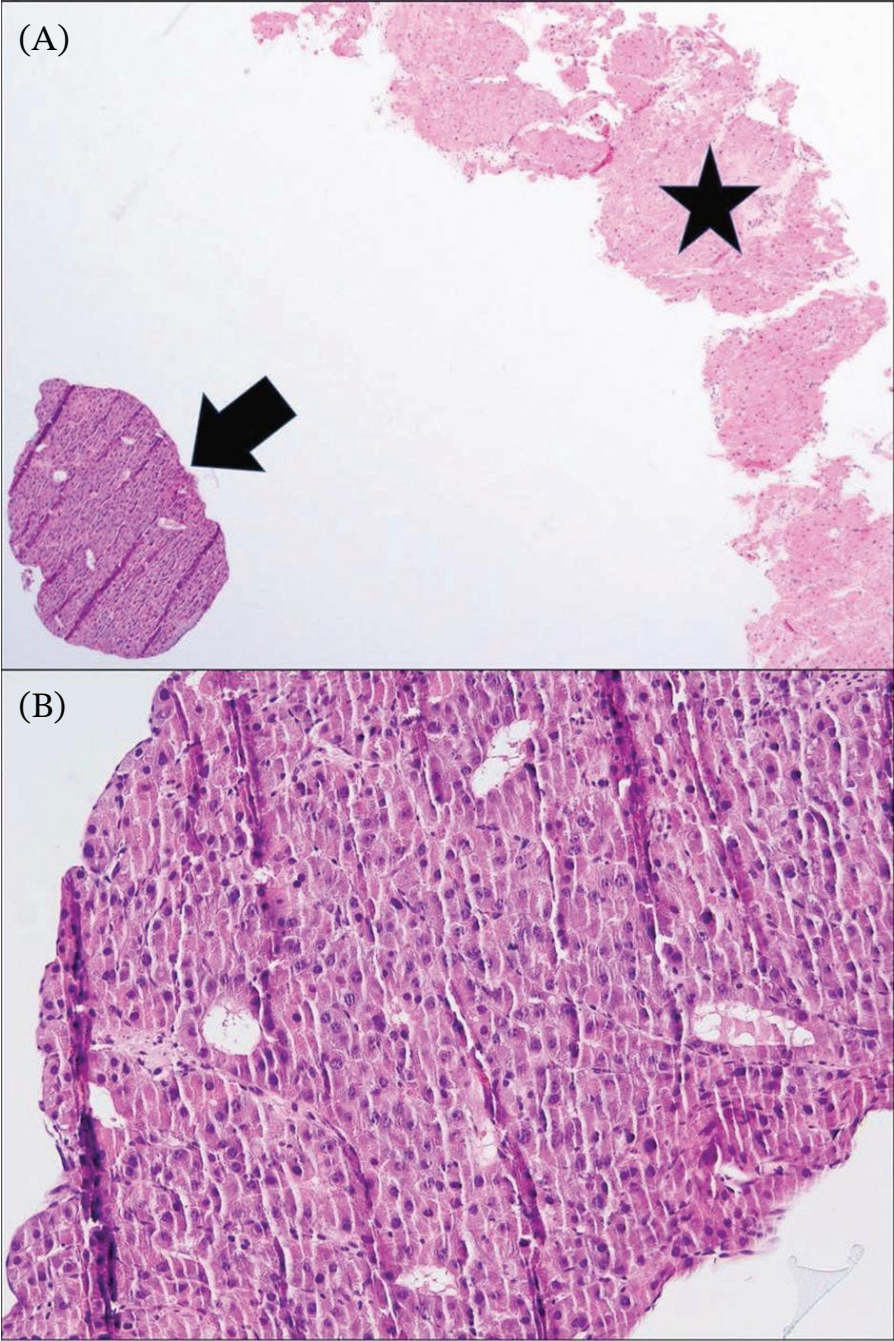
Images from H&E‐stained slides. (A) (40x magnification) shows pink myocardial tissue on the right (black star) with a fragment of malignant tissue on the left (black arrow). (B) (200x magnification of the malignancy) shows that the malignant cells grow in cords and acinar structures with eosinophilic cytoplasm, round nuclei, and visible nucleoli, morphologically consistent with hepatocellular carcinoma.

Prior to this presentation, the patient had already received two sessions of dual immunotherapy with durvalumab and tremelimumab. Following biopsy‐confirmed diagnosis of metastatic HCC, he received an additional session of this regimen after discharge. At 6‐month follow‐up, he reported symptomatic improvement and remained clinically stable, after which he also received palliative radiation therapy. However, his disease course subsequently worsened; hospice care was discussed with the patient approximately a year postdiagnosis, and he passed away 4 months thereafter.

## 3. Discussion

Cardiac metastases are significantly more common than primary cardiac neoplasms, most frequently arising from breast and bronchogenic carcinomas, leukemia, lymphoma, and sarcoma. [4, 5] HCC, although the most common primary liver malignancy, the sixth most common cancer worldwide, and the third leading cause of cancer‐related death, metastasizes to the heart with only an estimated incidence of 5%–10% [1] [2]. When cardiac involvement does occur, it typically affects the RA via direct tumor extension through the IVC [6]. Isolated RV metastasis without IVC or right atrial involvement, as seen in our patient, is exceedingly rare and sparsely reported in the literature. In the largest systematic review to date, of cardiac metastases in HCC, Fan et al. identified 80 published cases from the 1950s to 2019, with the RA being the most common site (52%) and isolated RV involvement accounting for only 14% of cases. [3] Zhang et al. similarly identified 13 cases of isolated RV metastasis between 1980 and 2018, the majority with myocardial infiltration and various patterns of RV obstruction [6]. The absence of IVC or atrial involvement in our patient, combined with FDG‐avid mediastinal and hilar lymphadenopathy, suggests hematogenous and possibly lymphatic routes of dissemination.

Diagnosing cardiac metastasis requires a high index of suspicion, as symptoms including dyspnea, chest discomfort, arrhythmia, and signs of right heart failure are nonspecific and overlap with many cardiac conditions. Multimodal imaging plays a crucial role in the diagnosis, as in our case [7]. TTE identified the mass and characterized its morphology; cardiac MRI delineated its extent and infiltrative behavior; and FDG‐PET distinguished metabolically active malignancy from thrombus or benign lesion, a distinction that directly shaped the decision to pursue tissue biopsy. Although FDG‐PET is not routinely employed in HCC surveillance given its low sensitivity and variable FDG avidity, its targeted application here was diagnostically pivotal [7–9].

Histopathological confirmation remains essential when management depends on tissue diagnosis particularly in patients with a history of malignancy where treatment decisions, including systemic therapy eligibility hinge on confirmed recurrence. Transvenous endomyocardial biopsy (EMB) offers a minimally invasive, real‐time image‐guided alternative to surgical resection, with relatively low complication rates and adequate diagnostic yield [10]. Thus, it demonstrates its utility in complex cardiovascular cases while preventing surgery [11]. In our case, biopsy via the right IJ transvenous approach provided safe and effective tissue acquisition leading to definitive confirmation, avoiding the morbidity of surgical resection, and directly enabled resumption of systemic therapy. This approach, although not universally applicable, is particularly valuable in patients with significant comorbidities or when surgical risk is high. Only a limited number of cases in the literature have employed this technique for intracardiac mass diagnosis [12, 13]. Our case adds to the limited but growing evidence supporting the utility of transvenous biopsy in diagnosing intracardiac masses, specifically large, nonmobile, isolated RV masses. However, the diagnostic yield of the transvenous approach may differ based on factors like tissue characteristics and tumor location, and methods like surgical resection or percutaneous CT‐guided biopsy have been considered in such scenarios [14, 15].

An additional distinctive feature of this case is its temporal course. HCC typically metastasizes within the first few years following diagnosis, yet our patient developed an isolated cardiac lesion 5 years after TACE, without hepatic recurrence detected until the time of cardiac presentation. This prolonged latency is exceptional and highlights the importance of sustained vigilance for extrahepatic metastasis, even in patients considered to be in remission.

Regarding prognosis and management, no standardized treatment protocols exist for cardiac metastasis from HCC given its rarity. Management is largely palliative, encompassing systemic therapy, and in select cases, surgical resection or radiation. Fan et al. in their review reported the highest overall survival in RV metastasis cases (45.5%) among cardiac sites, with best outcomes in patients undergoing surgery combined with multimodal therapy [3]. Zhang et al. reported that prognosis was generally poor with survival up to 9 months in isolated RV cases, and treatment focused on symptom relief [6]. The clinical course of our patient is consistent with this literature: despite prompt histopathologic diagnosis enabling resumption of systemic dual immunotherapy with durvalumab and tremelimumab, and subsequent palliative radiation therapy, the patient′s overall survival was approximately 18 months from the time of cardiac metastases diagnosis. This underscores a critical point that, although early and definitive diagnosis via transvenous biopsy meaningfully directed the therapeutic strategy and allowed the patient to pursue active treatment rather than empiric management, it did not alter the fundamentally poor prognosis associated with cardiac metastasis from HCC. In our case, it facilitated informed treatment decision‐making, appropriate goals‐of‐care discussions, and avoidance of unnecessary surgical intervention; outcomes that carry meaningful clinical value even in the palliative setting.

Because of the rarity of such cases, there are no standardized treatment protocols for cardiac metastases from HCC. Management is largely palliative and may include systemic therapy, radiation (rarely used because of cardiac toxicity), or surgical resection in select cases. Individualized, multidisciplinary decision‐making, integrating imaging, histopathology, systemic therapy, and goals‐of‐care discussions remains the cornerstone of management in this setting. To date, large‐scale clinical studies on HCC involving cardiac extension remain limited. Additional case reports and series are essential to better characterize clinical outcomes and guide the development of evidence‐based management strategies [16–18].

## 4. Conclusion

Isolated RV metastasis from HCC is exceedingly rare, with only a small number of histologically confirmed cases reported in the literature. This case illustrates the diagnostic and procedural utility and feasibility of transvenous biopsy in confirming intracardiac metastasis, even in anatomically challenging locations, while avoiding the morbidity of surgical resection. A multimodality imaging approach incorporating TTE, cardiac MRI, and FDG‐PET was essential in characterizing the mass and guiding the decision to pursue tissue diagnosis. Clinicians should maintain a high index of suspicion for cardiac metastasis in patients with known malignancy presenting with new cardiac symptoms, regardless of the interval since initial diagnosis. Although prognosis remains poor once cardiac metastasis is established, early and definitive diagnosis enables timely, individualized treatment planning and informed goals‐of‐care discussions, outcomes that remain clinically meaningful even in the absence of curative options.

## Author Contributions

**Mounika Reddy Vadiyala:** conceptualization, investigation, writing – original draft, writing – review and editing. **Sehajpreet Singh:** conceptualization, investigation, visualization, writing – review and editing. **Azka Naeem:** writing – review and editing. **Hakob Harutyunyan:** writing – review and editing. **Supraja Achuthanandan:** writing – review and editing. **David J. Epstein:** writing – review and editing. **Jasveen Kaur:** writing – review and editing. **Vijay Shetty:** supervision, writing – review and editing. **Simon Thein:** resources, writing – review and editing. **Gerald Hollander:** writing – review and editing. **Jacob Shani:** conceptualization, supervision, writing – review and editing.

## Funding

No funding was received for this manuscript.

## Disclosure

All authors have read and approved the final version of the manuscript. The corresponding author had full access to all of the data in this case report and takes complete responsibility for the integrity and accuracy of the case report.

## Ethics Statement

Ethical clearance was not required as it is a single anonymized case report with informed consent.

## Consent

Verbal informed consent was obtained from the patient for publication of this case report and accompanying images.

## Conflicts of Interest

The authors declare no conflicts of interest.

## Supporting information


**Supporting Information** Additional supporting information can be found online in the Supporting Information section. Data S1: CARE checklist of information to include when writing a case report.

## Data Availability

The authors confirm that the data supporting the case report are available within the article.
